# Durable recurrence-free survival after pneumonectomy for late lung metastasis from rectal cancer: case report with genetic and epigenetic analyses

**DOI:** 10.1186/s12885-015-1585-2

**Published:** 2015-08-01

**Authors:** Andrea Imperatori, Nicola Rotolo, Lorenzo Dominioni, Elisa Nardecchia, Maria Cattoni, Laura Cimetti, Cristina Riva, Fausto Sessa, Daniela Furlan

**Affiliations:** 1 Center for Thoracic Surgery, Department of Surgical and Morphological Sciences, University of Insubria, Ospedale di Circolo, Via Guicciardini, 9, Varese, 21100 Italy; 2 Section of Anatomic Pathology, Department of Surgical and Morphological Sciences, University of Insubria, Ospedale di Circolo, Via O. Rossi, 9, Varese, 21100 Italy

**Keywords:** Lung metastasis, Pneumonectomy, Rectal cancer metastasis, Long-term survival, Epigenetic factors

## Abstract

**Background:**

Treatment of pulmonary recurrence from colorectal cancer involving the main bronchus usually entails palliation using interventional bronchoscopy, because the prognosis is generally very poor. Surgical experience has clarified that in this setting pneumonectomy should only be performed in carefully selected patients showing favorable prognostic profiles (defined by low carcinoembryonic antigen serum levels pre-thoracotomy), solitary and completely resectable pulmonary metastasis, and long disease-free intervals. In the few long-term survivors after pneumonectomy for late-recurrent colorectal cancer, the disease has a relatively indolent metastatic course and genetic and epigenetic profiling may provide further insight regarding tumor evolution.

**Case presentation:**

We describe a rare case of late hilar-endobronchial and lymph nodal recurrence of rectal cancer, sequential to hepatic metastasectomy, that we successfully treated with pneumonectomy and chemotherapy (leucovorin, 5-fluorouracil and oxaliplatin regimen); the patient achieved 7-year relapse-free survival after lung metastasectomy and 24-year overall survival after primary rectal cancer resection. To our knowledge, this is the longest survival reported after sequential liver resection and pneumonectomy for recurrent colorectal cancer. In our case the primary rectal cancer and its recurrences showed identical immunohistochemical patterns. The primary rectal cancer and the matched metastases (hepatic, pulmonary and lymph nodal) demonstrated no *KRAS, NRAS, BRAF* and *PIK3CA* mutations, a microsatellite stable phenotype, and no tumor protein *p53* alterations or recurrent copy number alterations on chromosome 8. High genetic concordances between the paired primary tumor and metastases suggest that the key tumor biological traits remained relatively conserved in the three metastatic sites. Minor differences in gene specific hypermethylation were observed between the primary tumor and lung and nodal metastases. These differences suggest that epigenetic mechanisms may be causally involved in the microenvironmental regulation of cancer metastasis.

**Conclusion:**

The exceptionally long survival of the patient in our case study involving favorable clinical features was related to an excellent response to surgery and adjuvant chemotherapy; however, genetic or epigenetic factors that remain unidentified cannot be excluded as contributory factors. Our findings support the concept of a common clonal origin of the primary cancer and synchronous and late metastases, and suggest that aberrant DNA methylation may regulate tumor dormancy mechanisms.

## Background

The occurrence of sequential or concurrent liver and lung metastases after resection of colorectal cancer (CRC), with the exception of carefully selected patients, has traditionally been associated with poor long-term outcome [1–3]. For lung recurrence involving the proximal bronchi, the role of radical surgical cure remains uncertain, while palliation can be achieved by interventional bronchoscopy [4]. Because there have been few reports regarding patients with a history of hepatic metastasectomy for CRC who underwent pneumonectomy for the cure of a subsequent metastasis to the lung hilum [5–7], the clinical profile of patients who might benefit from pneumonectomy in this setting remains undefined [8, 9]. Moreover, metastases are seldom excised or biopsied in advanced-stage patients and there have been few studies that have focused on paired analysis of primary tumors and metastases for the purpose of tracing the tumor lineage during metastasization [10].

Here, we present a rare case of solitary hilar recurrence of rectal cancer with endobronchial extension, diagnosed at 17 years after primary rectal tumor resection and hepatic metastasectomy; the patient was treated with pneumonectomy and chemotherapy and has achieved an overall survival time of 24 years. In this patient, we performed a comprehensive study of the histologic, immunohistochemical, genetic and epigenetic profiles of the primary tumor and of its metastases to the liver, lung and lymph nodes.

## Case presentation

**Fig. 1:**
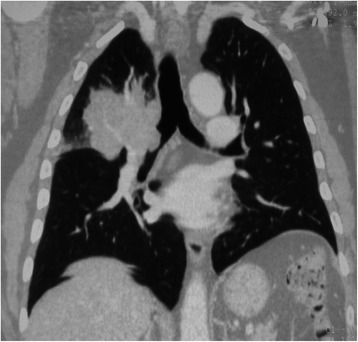
Chest Computed Tomography (CT). Chest CT scan coronal view showing 5-cm right hilar mass bulging into the main bronchus

**Fig. 2:**
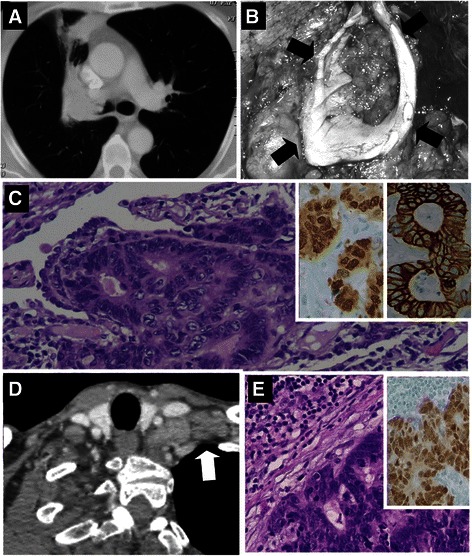
Chest CT details, histological and immunohystochemical studies. **a** Chest CT-scan axial view (lung window) showing a right hilar mass and post-obstructive collapse in the upper lobe anterior segment. **b** Close view of right pneumonectomy bronchial section margin (arrows); the main bronchus is obstructed by a polypoid tumor. **c** Lung metastatic adenocarcinomatous proliferation (hematoxylin & eosin staining, original magnification × 400); inset shows CDX-2 immunohistochemical staining (left), and CK20 positivity (right). **d** Chest CT-scan six months after right pneumonectomy, demonstrating enlarged left supraclavicular lymph nodes (arrow). **e** Lymph node metastasis with morphological features similar to Fig. 2c (hematoxylin & eosin staining, original magnification × 400); inset shows CDX-2 positivity

In 1990, a 40-year-old male underwent anterior resection of rectal adenocarcinoma (Dukes B2; preoperative carcinoembryonic antigen (CEA) serum level: 35 ng/mL), that was diagnosed synchronously with a 9-cm metastasis in liver segments V-VIII. After postoperative 5-fluorouracil based chemotherapy, the CEA serum level was slightly decreased (22 ng/mL); liver metastasis was markedly downsized and was radically resected. Follow-up revealed no evidence of residual disease until 2002; follow-up was then intentionally interrupted because the patient was considered to be free of disease. On March 2007, the patient developed dyspnea and cough, and a chest computed tomography (CT) scan revealed a 5-cm right hilar mass bulging into the main bronchus and infiltrating the upper lobe (Figs. 1 and 2a). Bronchoscopy demonstrated a neoplastic growth that was obstructing the right main bronchus 2 cm distal to the carina; biopsy revealed moderately differentiated adenocarcinoma, compatible with rectal cancer recurrence. Colonoscopy and CT of the abdomen and pelvis showed no other evidence of neoplastic disease, and the CEA serum level was normal (1 ng/mL). On April 2007, the patient was aged 57 years and was fit for major thoracic surgery. He was treated by means of right pneumonectomy and regional lymphadenectomy to obtain complete resection (Fig. 2b). Histology of the excised lung metastasis (Fig. 2c) revealed an adenocarcinomatous proliferation with morphological features overlapping those of primary rectal cancer and of previously resected hepatic metastasis. The primary cancer and all of the metastatic sites were characterized by extensive complex tubular gland formation with sparse centroglandular necrotic foci. Nuclei were moderately or highly atypical, elongated and stratified. The mitotic index ranged from 17 (in rectal cancer) to 24 × 10 high power fields (in metastatic sites). A mild peritumoral lymphocytic infiltrate (cluster of differentiation 3 positive) was present. Budding was absent and there was no lymphovascular invasion. The immunophenotype included caudal-type homeobox protein 2 and cytokeratin (CK) 20 (CK 20) positivity (Fig. 2c, inset), whereas thyroid transcription factor-1 and CK 7 were negative. The main bronchus resection margin was tumor-free. Of the 18 lymph nodes excised, 4 (all peribronchial) were metastatic. The early postoperative course was complicated by empyema that was successfully treated with antibiotics and drainage. Follow-up with CT scans of the chest and abdomen at 6 months after pneumonectomy revealed enlarged left supraclavicular lymph nodes (Fig. 2d). Biopsy of the latter revealed a metastatic adenocarcinomatous growth with a high mitotic index (14 × 10 high-power fields) and a morphological and immunohistochemical profile identical to that previously described (Fig. 2e). There were no other signs of relapse. Systemic chemotherapy (leucovorin, 5-fluorouracil and oxaliplatin regimen) was then initiated and continued until July 2008, resulting in complete clinical remission. Subsequent follow-up with semi-annual assessments of CEA level and total body CT and/or a positron emission tomography scans revealed no evidence of recurrence. At the last examination, in February 2015, the patient was relapse-free and enjoyed a good quality of life, at 7 years after pneumonectomy for lung metastasis and at 24 years after rectal cancer resection. Genetic and epigenetic profiles of the primary tumor and the three matched metastases (pulmonary, hepatic and lymph nodal) were assessed to verify the clonal origin of the four tumor samples and to characterize key biological traits during the tumor evolution. Tables 1 and 2 summarize the molecular results. Methylation specific-multiplex ligation dependent probe amplification (MS-MLPA) was performed by using the ME001 MS-MLPA tumor suppressor-1 kit and the ME002 MS-MLPA tumor suppressor-2 Kit (MRC-Holland, Amsterdam, The Netherlands) to simultaneously analyze the methylation status of 33 tumor suppressor genes and copy number alterations (CNA) of 53 genes [11]. The primary rectal carcinoma showed a high frequency of CNAs at multiple chromosome regions suggesting an unstable karyotype in this tumor. Clonal CNAs including chromosome gains at 7q, 9pq, 11pq, 13q, 19p and 20q were recurrent in the primary tumor and in the matched metastases, demonstrating a common origin of the four tumor samples (Table 1). Similarly, clonal gene specific hypermethylation was observed at adenomatous polyposis coli (APC) and cadherin 13 (CDH13) loci, while additional hypermethylated genes, namely *WT1, DAPK1, CHFR, PAX5* and *GATA5*, were found in the lung and in supraclavicular lymph node metastases (Table 2). Microsatellite instability (MSI) analysis, carried out using a pentaplex panel of monomorphic mononucleotide repeats (BAT25, BAT26, NR-21, NR-22 and NR-24) as previously reported [12], demonstrated the absence of MSI in all tumor samples. The Maldi-TOF mass spectrometry platform and the myriapod colon status kit (Diatech Pharmacogenetics, Jesi, Italy) were used to profile the patient’s four tumor samples, analyzing the 216 common hot-spot cancer mutations in the four major oncogenes involved in CRC pathogenesis (*KRAS, BRAF, PIK3CA* and *NRAS*). This analysis demonstrated no gene mutation in the primary rectal adenocarcinoma, and interestingly this genetic profile remained relatively well preserved in the three metastatic sites.

**Table 1:** Copy number alterations (CNAs) observed in the primary tumor and in the matched metastases

Chromosomal position	Primary rectal carcinoma	Hepatic metastasis	Lung metastasis	Lymph nodal metastasis
1p	+	+	-	+
2p	+	+	-	+
3p	+	+	-	-
7q	+	+	+	+
9p	+	+	+	+
11p	+	+	+	+
11q	+	+	+	+
12p	+	+	-	-
12q	+	+	-	-
13q	+	+	+	+
19p	+	+	+	+
20q	+	+	+	+

+: presence of CNA; -: absence of CNA

**Table 2:** Genetic and epigenetic alterations in the primary tumor and matched metastases

Tumor sample	Gene hypermethylation^a^	MSI^b^ status	*BRAF*	*KRAS*	*NRAS*	*PIK3CA*
**Primary rectal cancer**	***APC; CDH13;*** *WT1*	MSS	-	-	-	-
**Hepatic metastasis**	***APC; CDH13***	MSS	-	-	-	-
**Lung metastasis**	***APC; CDH13;*** *WT1; DAPK1; CHFR; GATA5*	MSS	-	-	-	-
**Lymph nodal metastasis**	***APC; CDH13;*** *PAX5; DAPK1; CHFR; GATA5*	MSS	-	-	-	-

^a^List of hypermethylated genes; clonal gene hypermethylations are shown in bold font. *APC* adenomatous polyposis coli, *CDH13* cadherin 13

^**b**^*MSI* microsatellite instability. MSS: microsatellite stable. -: absence of BRAF, KRAS, NRAS, PIK3CA mutations assayed using the myriapod colon status panel

## Discussion

The decision regarding surgical treatment is critical when an isolated lung metastasis can only be radically resected using pneumonectomy, as was true in the present case. Following sequential surgical resection of the hepatic and pulmonary recurrences from CRC, the following factors are predictive of long-term survival and can help in addressing surgical strategies: 1) low CEA serum level pre-thoracotomy [7]; 2) solitary pulmonary recurrence after a long disease-free interval [3, 7]; 3) complete resection of lung recurrence after hepatic metastasectomy [5]; and 4) responsiveness to chemotherapy [2, 5]. Clearly, aggressive treatment of sequential recurrences can only be beneficial for carefully selected patients. Accordingly, the management of a hilar-endobronchial recurrence from CRC should be individualized after considering all endoscopic and surgical options. In the case presented here, potentially radical resection in a fit-for-surgery patient with a long-term clinical history suggestive of a relatively indolent rectal cancer (associated with a normal CEA serum level) lead to the indication of pneumonectomy for the treatment of an isolated recurrence of rectal cancer involving the main bronchus. We did not perform right upper lobe sleeve resection because at intraoperative evaluation this procedure was inadequate in achieving local radical excision of the pulmonary metastasis and lymph nodes. To our knowledge, the survival time of our case is the longest so far reported after sequential liver resection and pneumonectomy for recurrent CRC [7].

Extensive genetic and epigenetic profiling of the primary rectal adenocarcinoma allowed the characterization of the key biological traits of the tumor and comparison with the recent results of CRC molecular characterization [13]. According to this comprehensive classification, the primary cancer reported in the present case may probably be designated as a non-hypermutated tumor, because we did not find any of the hot-spot gene mutations commonly observed in CRC. Moreover, it did not exhibit the presence of MSI or a cytosine-phospho-guanine island methylator phenotype. As expected, this non-hypermutated tumor had a large number of CNAs including several previously well-defined chromosomal and sub-chromosomal changes in CRC, such as gains of 7q, 11p, 13q and 20q. Remarkably, the tumor did not exhibit alterations in tumor protein *p53* (TP53) or recurrent CNAs on chromosome 8 that are the most frequent events in highly aggressive cancers exhibiting copy number genomic instability [13]. Finally, a key feature of the primary rectal adenocarcinoma was *APC* and *CDH13* methylation, suggesting an aberrant activation of the wingless signaling pathway that is considered a nearly ubiquitous event in CRC [13].

Comparison between the primary cancer and the matched metastases demonstrated high genetic concordances and minor differences among samples; this was in agreement with the current literature, supporting the concept that concordance is dominant during the metastatic process and reflects the common clonal origin of primary cancer and metastases [10]. Many studies have suggested that the primary tumor site is also the location where cancer cells obtain their metastatic potential [10]. They reach secondary organs through the circulatory system, and display dormancy, apoptosis or proliferation. According to this hypothesis, when the dormant cells receive some specific signals, metastasis will occur [14]. This model is supported by the similarities in gene expression signatures observed between metastases and their corresponding primary tumors in several different types of cancer including breast, pancreatic, CRC and prostate [15]. Recent evidence has suggested that epigenetic mechanisms, such as aberrant DNA methylation and microRNAs, may regulate the long-term commitment of disseminated tumor cells to quiescence while retaining growth potential [15, 16]. In the case reported here, the different methylation profiles of lung and lymph nodal metastases, as compared with the primary cancer and the liver metastasis, are consistent with this hypothesis. The unexpected finding of a normal CEA level at the time of lung recurrence and subsequent supraclavicular lymph node recurrence, and the fact that the genetic signature of the primary tumor and matched metastases remained stable, could be explained by epigenetic mechanisms. The latter are well known to modulate the expression of adhesion molecules during the metastatic process [17, 18]. Interestingly, the sensitivity of the CEA test has been shown to depend on the site of CRC metastasis; in a large multicenter study, the test was reported to be grossly insensitive for solitary lung recurrences, because these were detectable using CEA in only 15 % of cases [19].

## Conclusions

Cancer survival is in part related to the natural biology of slow-growing tumors. However, here we presented the case of a patient with pulmonary and lymph nodal metastases that occurred many years after primary tumor removal; these metastases exhibited morphological features of aggressive growth, including marked atypia, high mitotic index and tumor necrosis. The extensive genetic and epigenetic profiling of the primary rectal cancer and of the matched metastases demonstrated the absence of *KRAS, NRAS, BRAF* and *PIK3CA* mutations, a microsatellite stable phenotype; in addition, there were no TP53 alterations and no recurrent CNAs on chromosome 8. Our findings support the concept of the common clonal origin of the primary cancer and of the synchronous and late metastases, suggesting that the key tumor biological traits remained quite conserved and that epigenetic mechanisms may be causally involved in the microenvironmental regulation of cancer metastasis. The exceptionally long survival time of the current case was probably related to an excellent response to surgery and adjuvant chemotherapy; however, it might also be associated with yet to be identified genetic or epigenetic factors involved in the change from dormancy to the activation of metastasis.

Our data are preliminary and need to be confirmed, but they suggest the importance of future genome-scale analyses of methylomes and microRNA expression in comparing primary tumors and matched metastases.

## Consent

Written informed consent was obtained from the patient for publication of this case report and any accompanying images. A copy of the written consent is available for review by the Editor of this journal.

## Abbreviations

CRC: Colorectal cancer
CT: Computed tomography
CNA: Copy number alterations
MSI: Microsatellite instability
MSS: Microsatellite stable
CEA: Carcinoembryonic antigen
CK: Cytokeratin
MS-MLPA: Methylation specific-multiplex ligation dependent probe amplification
APC: Adenomatous polyposis coli
CDH13: Cadherin 13
TP53: Tumor protein *p53*

## References

[1] Rees M, Tekkis PP, Welsh FK, O’Rourke T, John TG (2008). Evaluation of long-term survival after hepatic resection for metastatic colorectal cancer: a multifactorial model of 929 patients. Ann Surg.

[2] Sourrouille I, Mordant P, Maggiori L, Dokmak S, Lesèche G, Panis Y (2013). Long-term survival after hepatic and pulmonary resection of colorectal cancer metastases. J Surg Oncol.

[3] Miller G, Biernacki P, Kemeny NE, Gonen M, Downey R, Jarnagin WR (2007). Outcomes after resection of synchronous or metachronous hepatic and pulmonary colorectal metastases. J Am Coll Surg.

[4] Fournel C, Bertoletti L, Nguyen B, Vergnon JM (2009). Endobronchial metastases from colorectal cancers: natural history and role of interventional bronchoscopy. Respiration.

[5] Neeff H, Hörth W, Makowiec F, Fischer E, Imdahl A, Hopt UT (2009). Outcome after resection of hepatic and pulmonary metastases of colorectal cancer. J Gastrointest Surg.

[6] Shah SA, Haddad R, Al-Sukhni W, Kim RD, Greig PD, Grant DR (2006). Surgical resection of hepatic and pulmonary metastases from colorectal carcinoma. J Am Coll Surg.

[7] Regnard JF, Grunenwald D, Spaggiari L, Girard P, Elias D, Ducreux M (1998). Surgical treatment of hepatic and pulmonary metastases from colorectal cancers. Ann Thorac Surg.

[8] Pfannschmidt J, Hoffmann H, Dienemann H (2010). Reported outcome factors for pulmonary resection in metastatic colorectal cancer. J Thorac Oncol.

[9] Rotolo N, De Monte L, Imperatori A, Dominioni L (2007). Pulmonary resections of single metastases from colorectal cancer. Surg Oncol.

[10] Gao D, Li S (2013). Biological resonance for cancer metastasis, a new hypothesis based on comparisons between primary cancers and metastases. Cancer Microenviron.

[11] Furlan D, Sahnane N, Bernasconi B, Frattini M, Tibiletti MG, Molinari F (2014). APC alterations are frequently involved in the pathogenesis of acinar cell carcinoma of the pancreas, mainly through gene loss and promoter hypermethylation. Virchows Arch.

[12] Furlan D, Carnevali IW, Bernasconi B, Sahnane N, Milani K, Cerutti R (2011). Hierarchical clustering analysis of pathologic and molecular data identifies prognostically and biologically distinct groups of colorectal carcinomas. Mod Pathol.

[13] Muzny DM, Bainbridge MN, Chang K, Dinh HH, Drummond JA, Fowler G, et al. Comprehensive molecular characterization of human colon and rectal cancer. Nature. 2012;487:330–7. 10.1038/nature11252 PMC3401966 22810696

[14] Rotolo N, Dominioni L, De Monte L, Conti V, La Rosa S, Imperatori A (2013). Metastasis at a tracheostomy site as the presenting sign of late recurrent breast cancer. Head Neck.

[15] Zhang Y, Yang P, Wang XF (2014). Microenvironmental regulation of cancer metastasis by miRNAs. Trends Cell Biol.

[16] Bedi U, Mishra VK, Wasilewski D, Scheel C, Johnsen SA (2014). Epigenetic plasticity: a central regulator of epithelial-to-mesenchymal transition in cancer. Oncotarget.

[17] Cock-Rada A, Weitzman JB (2013). The methylation landscape of tumour metastasis. Biol Cell.

[18] Hammarstrom S (1999). The carcinoembryonic antigen (CEA) family: structure suggested functions and expression in normal and malignant tissues. Semin Cancer Biol.

[19] Moertel CG, Fleming TR, MacDonald JS, Haller DG, Laurie JA, Tangen C (1993). An evaluation of the carcinoembryonic antigen (CEA) test for monitoring patients with resected colon cancer. JAMA.

